# Perioperative high dose rate brachytherapy in head and neck cancers: case report and review of clinical application

**DOI:** 10.1259/bjrcr.20200158

**Published:** 2021-04-12

**Authors:** Amit Bahl, Roshan K Verma, Naresh K Panda, Arun S Oinam, Jerry R John, Satinder Kaur, Pramod Kumar, Sushmita Ghoshal, Gaurav Trivedi, Jaimanti Bakshi

**Affiliations:** 1 Department of Radiotherapy, Postgraduate Institute of Medical Education & Research, Chandigarh, India; 2 Department of Otolaryngology, Postgraduate Institute of Medical Education & Research, Chandigarh, India; 3 ,Department of Plastic Surgery, Postgraduate Institute of Medical Education & Research, Chandigarh, India

## Abstract

Perioperative high dose rate brachytherapy is a radiotherapy treatment technique which involves intraoperative insertions of brachytherapy catheters into the tumor bed during the surgical resection followed by treatment in the post-operative period. We report here two cases to highlight its use in the primary treatment and reirradiation of head and neck cancers.

## Introduction

The first reported application of using a radioisotope to treat a malignancy intraoperatively is credited to Robert Abbe in the year 1910.^1^ This technique in time evolved into the discipline of Brachytherapy and it has established itself as an important treatment modality over the last century. Brachytherapy using iridium interstitial implants has been practised in head and neck cancers. It has also been combined with surgical resection of tumors with treatments delivered either intraoperatively or perioperatively. Perioperative high dose rate brachytherapy (PHDRB) is a technique which involves intraoperative insertions of brachytherapy catheters in the tumor bed at time of surgical resection followed by a fractionated brachytherapy treatment in the perioperative period.^2^ It combines the advantages of a highly conformal radiation dose delivery with a clear visualization and demarcation of the tumor bed at the time of surgical dissection. This coupled with incorporation of CT-based treatment planning gives it a high level of treatment delivery precision. Perioperative brachytherapy is an established technique of delivering radiotherapy treatments in sarcomas and its application has been reported in pancreatic cancers.^3^ Its use in head and neck cancers has been infrequent due to the complex regional anatomy, surrounding vascular and nervous tissues, multitude of organs at risk and the invasive nature of the procedure. PHDRB can be used in head and neck cancers to primarily irradiate the tumor bed to a very high dose of radiation as a single modality or combined with external beam radiotherapy in both the primary treatment and for reirradiation of recurrent cancers.^4^ We report here two cases highlighting the applications of perioperative brachytherapy in these clinical situations.

## Clinical presentation

**Figure 1.**
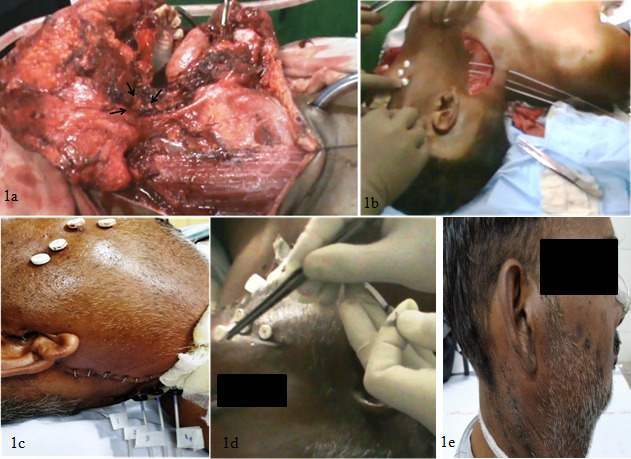
(a)Tumor bed with gross tumor removed and high risk area marked with arrows (b) Intraoperative brachytherapy catheter insertion in the tumor bed (c) Post operative status with catheters in the tumor bed (d) Catheter removal after completion of treatment (e) Two weeks post brachytherapy treatment

**Figure 2. F2:**
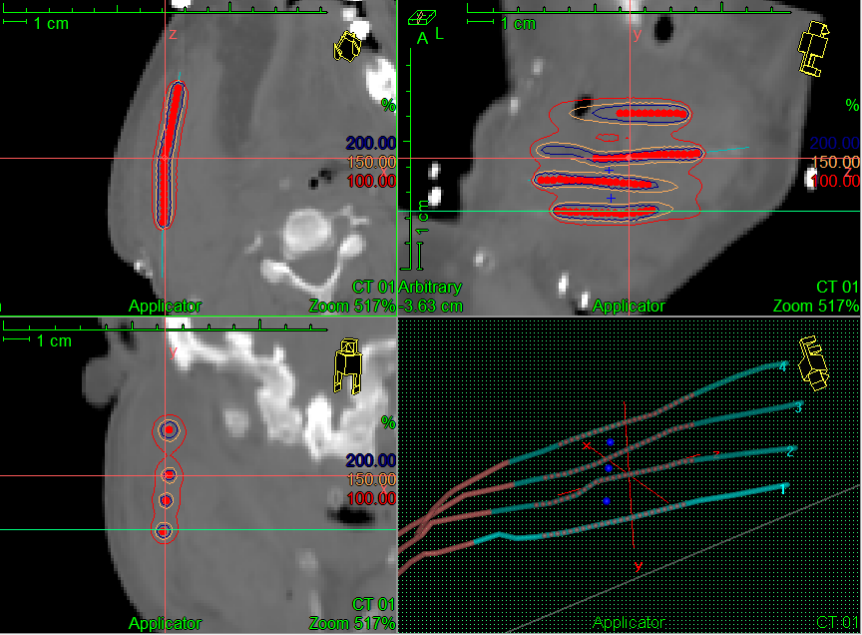
CT cross section image from Treatment Planning System showing reconstructed catheters

The first case was a 45 years male who presented with a non-healing ulcer in posterior aspect of the right oral cavity of 2 months duration. The patient was evaluated in the multidisciplinary head and neck cancer tumor board of our institute. Examination revealed a right level Ib lymph node of 2 × 2 cm which was firm mobile, non-tender. Local examination of the oral cavity showed an ulceroinfilitrative growth 6 × 4 cm in size involving the right retromolar trigone region. Biopsy was suggestive of squamous cell carcinoma. Patient was diagnosed as a carcinoma of the right retromolar trigone cT3N1M0 (Stage III). The patient was planned for surgery with post-operative radiotherapy using a combination of perioperative and external beam radiotherapy in view of anticipated close margins. He underwent a wide local excision and right inferior maxillectomy along with right modified neck dissection Type III with right segmental mandibulectomy ( Figure 1a). Frozen section done during surgery after maximal possible resection showed positive surgical margins in the retromolar area. Four interstitial brachytherapy catheters were inserted 1.5 cm apart into the tumor bed (Figure 1b). The catheters were secured to the tumor bed with absorbable sutures. Surgical reconstruction was done using a Deltopectoral and Pectoralis Musculocutaneous flap. The patient underwent a treatment planning CT scan on the third post-operative day with a CT slice thickness of 2.5 mm. Brachytherapy planning (Figure 2) was done on Oncentra brachytherapy planning system 4.3 (Elekta, Stockholm, Sweden). A dose homogenity index ([ V_100_ - V_150_] / V_100_) of 0.60 was achieved on planning (V_100_ & V_150_ represent tissue volume encompassed by 100% and 150% prescription isodose). Brachytherapy treatment was started from the fourth post-operative day on microSelectron HDR (Elekta, Stockholm,Sweden) and treatments were delivered twice a day at 6-hourly intervals for a total of 7 fractions of 3 Gy each Figure 1c. Brachytherapy catheters were removed on last day of treatment Figure 1d. The patient was reviewed for proper scar healing after surgical sutures were removed and started on external beam radiotherapy to a dose of 50 Gy delivered over 5 weeks. The patient was disease free at last follow-up at 1 year. This case highlights the use of PHDRB for radiotherapy dose escalation and increasing the surgical margins in the primary treatment of these cancers.

**Figure 3.**
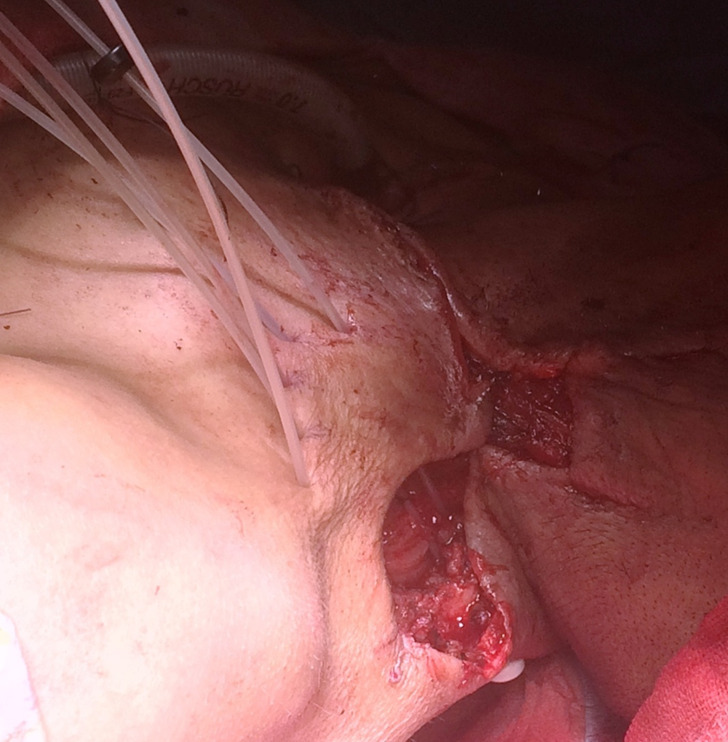
Intraopertaive brachytherapy catheter insertion in the tumor bed in the level V nodal region after resection of recurrent nodal disease

The second patient was a 51-year-old male diagnosed with Carcinoma of the Base of Tongue cT3N2bM0 (Stage IVA). He had received radical radiotherapy to a dose of 70 Gy over 35 fractions delivered in 7 weeks along with concurrent Inj. Cisplatin 100 mg/ m^2^ D1, 22 in the year 2014. The radiation was delivered using conventional radiotherapy with two parallel opposed head and neck treatment portals. He presented with a mass in the right lower neck of 2 month duration in 2017. On examination, there was left level Vb 5 × 5 cm lymph node mass. It was firm and fixed to underlying structures. Contrast-enhanced CT scan was suggestive of infilitration into adjoining soft tissues and biopsy revealed a squamous cell carcinoma. Patient was staged as a recurrent carcinoma base of tongue rT0N2bM0 (rStage IVA). The patient was planned for surgical excision with modified neck dissection and reirridation using perioperative brachytherapy. The indications for perioperative brachtherapy were extracapsular spread of disease with infiltraion into the sternocleidomastoid muscle and an anticipated doubtful R0 resection. The patient underwent modified neck dissection with excision of nodal mass. Five brachytherapy catheters were inserted over tumor bed with intercatheter spacing of 1.5 cm (Figure 3)[Fig F2 F4]. The patient was planned for a brachytherapy dose of 30 Gy delivered in 12 fractions at 2.5 Gy per fraction.^5^ A dose homogeneity index of 0.67 was achieved for the brachytherapy treatment plan. The treatments were delivered twice a day at 6-hourly intervals. The patient was disease free at 9 months follow-up. This case demonstrates the use of PHDRB in reirradiation of recurrent head and neck cancers.

## Discussion

**Figure 4. F4:**
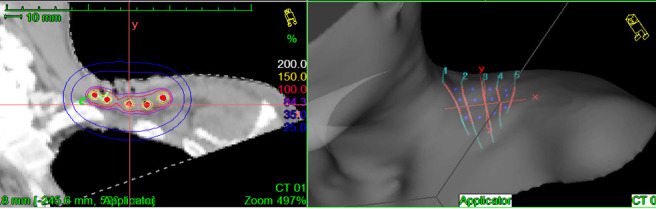
CT cross section image showing brachytherapy catheters with dose distribution

PHDRB exemplifies the true interdisciplinary management of cancers by integrating brachytherapy with surgery. The ‘*tumor bed effect’* theory explains the rationale of delivering a high dose of radiation in the immediate perioperative period. It alters the interaction of the microscopic residual disease cells within the tumor bed with the normal host tissues and prevents their reimplantation and subsequent local recurrences. Improved local control in turn lead to decreased local and distant failures and improved overall survival.^6^ Reducing the treatment-related toxicity is another argument for the practice of brachytherapy, as it confines the radiation dose to a small area with a rapid dose fall off in surrounding structures. Concurrent chemoradiation is considered the standard of care in management of locally advanced head and neck cancers. Significant Grade 3 toxicity ranging from 21 to 38% has been reported with the use of concurrent chemoradiotherapy protocols in head and neck cancers. Grade 3 toxicity upto 39% has been reported with reirradiation using external beam techniques.^7^ One of the ways to reduce this treatment associated toxicity is to reduce the volume of irradiation by integrating brachytherapy into the treatment protocols.The isodose distribution of PHDRB implants Figure 4 show the high degree of conformity achieved within the treatment area with minimal dose to the surrounding critical organs. Gaztanaga et al have shown perioperative high dose rate brachytherapy to have equivalent treatment outcomes when compared to wide field radiotherapy with 5 year locoregional control rates from 60.9 to 79.4%.^4^

Non-nasopharangeal head and neck cancers are a locoregional disease with reported recurrence rates as high as 50% after curative treatment.^8^ Reirradiation has been effectively used to manage recurrent head and neck cancers^6^ and PHDRB is an excellent modality to be used in this indication.Single plane PHDRB implants can deliver a high targeted dose to positive margins and can also increase the surgical margins by 1–1.5 cm thereby improving the local control.^9^ An added advantage of brachytherapy implants is that they are not affected by organ motion or respiratory movements.

Being an invasive treatment modality, perioperative brachytherapy needs a careful patient selection when being implemented in clinical practice. Patient selection for PHDRB can be aided by dividing patients requiring brachytherapy for reirradiation or those with inadequate surgical margins at difficult resection sites or those with adequate surgical margins as a means of dose escalation.^10^ To help patient selection, the University of Navarre predictive model can also be used which divides patients into low risk, intermediate, high and very high risk categories.^11^ Preplanning with the surgical team is useful and should also review the reconstruction procedure and type of surgical flaps to be used. Identifying the feeding vasculature for the surgical flap can prevent unintended irradiation to the flap vasculature and have implications on flap viability. Tumor location may be the limiting factor for selecting patients in head and neck cancers and PHDRB should be done in a site which allows easy catheter entry and exit without undue bending or knicking of catheters. A vast majority of implants in head and neck will be single plane with an aim to irradiate only the tumor bed as a target area. Table 1 gives the dose schedules of PHDRB reported in head and neck cancers. The GEC-ESTRO guidelines recommend restricting the individual dose fraction between 3 and 4 Gy per fraction for primary brachytherapy treatment.^9^

**Table 1.** Dose schedules in perioperative brachytherapy in head and neck cancers

Author	Indication	Brachytherapy Dose Two fractions(#) per day 6 h apart)	Dose of external beam radiotherapy (4–5 weeks after Surgery)
	REIRRADIATION		
Rudzianskas V et al^5^	-	2.5 Gy x 12 #	-
Gaztañaga M et al^4^	R0 resection R1 resection	4 Gy x 8 # 4 Gy x 10 #	- -
	NON-REIRRADIATION		
Martínez-Monge R^2^	R0 resection R1 resection R2 resection	4 Gy x 4 # 4 Gy x 6 # 4 Gy x 8 #	45 Gy / 25# / 5 weeks 45 Gy/25#/ 5 weeks 45 Gy/25#/ 5 weeks
Teudt IU et al^12^	-	2.5 Gy x 10–12#	40–63 Gy

Periopertive high dose rate brachytherapy alone after R0 resection has been associated with 9 year cancer-specific survival rate of 47.9%.^4^ Martinez et al reported the 4 year local control rate and overall survival were 85.6 and 46.4%, respectively in head and neck cancers treated with reirradiation using PHDRB.^13,14^ Treatment-related acute side-effects include bleeding, fistula, graft failure, delayed wound healing. Late morbidity can occur in the form of fibrosis, soft tissue necrosis and osteoradionecrosis. Overall, high grade toxicity has been reported to be between 15 and 69% in single modality procedures and 2.8–30.5% in combined modality procedures.^15^

This case report demonstrates the clinical application of PHDRB in head and neck cancers and can be considered as one on the treatment options in patients with high risk margins or where reirradiation is being planned after surgical resection.

## Learning points

1. PHDRB allows brachytherapy catheter placement in anatomical regions not easily accessible to conventional interstitial brachytherapy, since catheters are placed intraoperatively under direct tumor bed visualization.
2. Overall treatment time can be reduced by integration of PHDRB with external beam radiotherapy.
3. It is a versatile treatment to be used for increasing the surgical margins or for dose escalation in primary and recurrent head and neck cancers.
